# Remimazolam-based anesthesia in sepsis patients: a retrospective cohort study of hemodynamic stability and postoperative outcomes

**DOI:** 10.3389/fmed.2026.1838670

**Published:** 2026-07-01

**Authors:** Hyunyoung Seong, Sejong Jin, Yeon Jae Song, Woohyun Park, Hyun Ah Lee, Jang Eun Cho

**Affiliations:** 1 Department of Anesthesiology and Pain Medicine, Anam Hospital, Korea University College of Medicine, Seoul, Republic of Korea; 2 Department of Neuroscience, Korea University College of Medicine, Seoul, Republic of Korea; 3 Department of Anesthesiology and Pain Medicine, Korea University College of Medicine, Seoul, Republic of Korea

**Keywords:** hemodynamic stability, postoperative outcomes, remimazolam, sepsis, septic shock

## Abstract

**Background:**

Patients with abdominal sepsis or septic shock undergoing urgent source-control surgery are highly susceptible to anesthetic-induced hypotension, often requiring vasopressors that may stabilize mean arterial pressure (MAP) but worsen microcirculatory perfusion. This retrospective single-center study evaluated whether remimazolam-based general anesthesia reduces perioperative vasopressor burden and improves hemodynamic stability and postoperative outcomes compared with propofol induction followed by volatile maintenance.

**Methods:**

In this study, 220 consecutive Sepsis-3–defined patients who underwent abdominal surgery between January 2021 and June 2025 were analyzed, using a prospectively maintained database. Patients were categorized as propofol/volatile anesthesia (Group C, *n* = 116) or remimazolam induction and maintenance (Group R, *n* = 104). The primary outcome was the Vasopressor-Inotrope Score (VIS) at postoperative intensive care unit (ICU) admission; secondary outcomes included intraoperative hemodynamics across six time points, change in Sequential Organ Failure Assessment at 24 h, ICU/hospital length of stay (LOS), mortality, and complications.

**Results:**

In a matched cohort (87 per group), VIS at ICU admission was lower with remimazolam (*P* = 0.006), accompanied by shorter ICU LOS (*P* = 0.008) and hospital LOS (*P* = 0.012), lower in-hospital mortality (*P* = 0.007), and fewer hospital-acquired infections (*P* = 0.048), with no differences in mechanical ventilation duration, 30-day mortality, acute kidney injury, acute respiratory distress syndrome, delirium, major complications, or ICU readmission. Mixed-effects models showed more favorable intraoperative systolic blood pressure/MAP and heart-rate trajectories with remimazolam (significant group-by-time interactions).

**Conclusion:**

Remimazolam-based anesthesia was associated with greater hemodynamic stability and reduced postoperative vasopressor needs, translating to lower resource utilization and in-hospital mortality in septic surgical patients undergoing surgery, supporting prospective trials to test causality.

## 1 Introduction

Abdominal sepsis remains one of the most challenging surgical emergencies (1, 2) and is associated with substantial morbidity and mortality despite advances in surgical source control, critical care, and perioperative management (3, 4). Effective source control is the cornerstone of treatment and frequently requires urgent abdominal surgery (2, 5). However, patients with abdominal sepsis often present with profound physiological derangements, including sepsis-associated vasoplegia, relative hypovolemia resulting from capillary leak and fluid shifts, myocardial dysfunction, and evolving multiple-organ dysfunction (5–7). These pathophysiological abnormalities reduce cardiovascular reserve and increase susceptibility to anesthetic-induced hypotension and hemodynamic instability during emergency surgery (8, 9).

Maintenance of adequate tissue perfusion is a central goal during anesthetic management of patients with sepsis. Although mean arterial pressure (MAP) is widely used as a hemodynamic target, restoration of systemic blood pressure alone does not necessarily ensure adequate intestinal oxygen delivery (10–13). Sepsis-associated endothelial injury, microcirculatory dysfunction, vasoplegia, and coagulation abnormalities may impair regional perfusion despite apparently acceptable macrocirculatory parameters (14, 15). Excessive vasopressor administration may further exacerbate regional blood-flow maldistribution, particularly within the splanchnic circulation (16–18). Therefore, anesthetic strategies that minimize cardiovascular depression and reduce vasopressor requirements may be clinically advantageous in patients undergoing source-control surgery for abdominal sepsis.

Propofol remains the most commonly used intravenous anesthetic agent for induction and maintenance of general anesthesia. However, its use is frequently associated with dose-dependent vasodilation, myocardial depression, and hypotension, effects that may be particularly detrimental in patients with sepsis (9). Remimazolam is a novel ultra-short-acting benzodiazepine that undergoes rapid metabolism by tissue esterases and has emerged as a potential alternative intravenous anesthetic agent. Previous studies in various surgical populations have reported greater hemodynamic stability and a lower incidence of hypotension with remimazolam compared with propofol (19–21). Furthermore, recent reviews have suggested that remimazolam may offer favorable cardiovascular tolerance in critically ill patients while maintaining predictable recovery characteristics (9, 20, 22).

Despite these promising pharmacological properties, evidence regarding the perioperative use of remimazolam in patients with abdominal sepsis remains limited. Most available studies have been conducted in elective surgical populations, and the clinical significance of remimazolam-associated hemodynamic stability in septic patients undergoing emergency abdominal surgery remains uncertain. In particular, whether reduced anesthetic-induced cardiovascular depression translates into lower vasopressor requirements and improved postoperative outcomes has not been adequately investigated.

Therefore, we conducted a retrospective cohort study to compare remimazolam-based total intravenous anesthesia with conventional propofol induction followed by volatile anesthetic maintenance in patients undergoing emergency abdominal surgery for sepsis. We hypothesized that remimazolam-based anesthesia would be associated with reduced vasopressor requirements and improved perioperative hemodynamic stability, potentially contributing to more favorable postoperative outcomes in this high-risk population.

## 2 Materials and methods

### 2.1 Patients

This retrospective study was conducted using a prospectively maintained institutional database. We reviewed consecutive patients diagnosed with sepsis or septic shock according to the Sepsis-3 criteria who underwent abdominal surgery at Korea University Anam Hospital between January 1, 2021, and June 30, 2025, with an abdominal source of infection.

Among 238 eligible patients, the following were excluded: surgery aborted after induction of anesthesia because of severe hemodynamic instability (*n* = 3); receipt both volatile anesthetics and remimazolam during the same procedure (*n* = 4); incomplete medical records (*n* = 5); ongoing hospitalization at the time of data collection (*n* = 2); and multiple surgeries during the study period, in which case the first procedure was eligible (*n* = 4). After these exclusions, 220 patients were included in the final analysis and categorized according to the anesthetic agent administered.

### 2.2 Anesthetic management

The choice of anesthetic agent was determined by the attending board-certified anesthesiologist. The control group (Group C) received propofol for induction of anesthesia, followed by maintenance with a volatile anesthetic (sevoflurane or desflurane). The study group (Group R) received remimazolam for both induction and maintenance of general anesthesia.

In Group C, anesthesia was induced with an intravenous bolus of propofol (1.0–1.5 mg/kg) combined with a continuous infusion of remifentanil (0.05–0.1 μg/kg/min). Anesthesia was maintained with sevoflurane or desflurane at an age-adjusted minimum alveolar concentration of 0.8–1.0, together with continuous remifentanil infusion.

In Group R, remimazolam (Byfavo^®^, Hana Pharm Co., Seoul, Republic of Korea) was administered as a continuous infusion at 6 mg/kg/h for induction and 1–2 mg/kg/h for maintenance. In both groups, anesthetic dosing was titrated to maintain a bispectral index (BIS™, Medtronic, Dublin, Ireland) value between 40 and 60 to adjust depth of anesthesia, consistent with prior remimazolam anesthesia protocols (20, 23).

Standard intraoperative monitoring included electrocardiography, pulse oximetry, and noninvasive blood pressure measurement. Neuromuscular blockade was monitored using a peripheral nerve stimulator. After confirmation of loss of consciousness, rocuronium (0.6–1.0 mg/kg) was administered intravenously to facilitate tracheal intubation. During pneumoperitoneum, neuromuscular blockade was maintained to achieve a target train-of-four (TOF) count of 0–2. At the conclusion of surgery, neuromuscular blockade was reversed with sugammadex according to TOF count, and patients were subsequently transferred to the ICU.

In accordance with institutional protocols and current sepsis management guidelines, intraoperative MAP and HR were maintained within ±20% of baseline values. Norepinephrine was used as the first-line vasopressor for intraoperative hypotension. In patients receiving norepinephrine preoperatively for septic shock, the infusion was continued at the same dose upon arrival in the operating room. If hypotension persisted despite norepinephrine administration at doses up to 0.3 μg/kg/min, additional vasopressors, including epinephrine or vasopressin, were administered at the discretion of the attending anesthesiologist.

### 2.3 Data collection and outcomes

Demographic and baseline clinical variables included age, sex, body weight, height, body mass index (BMI), and ASA physical status. Preoperative comorbidities, including hypertension (HTN), diabetes mellitus (DM), chronic heart failure, and other relevant medical conditions, were recorded.

Septic shock was identified according to Sepsis-3 criteria. In this retrospective cohort, continuous norepinephrine infusion requirement was used as an operational surrogate marker for vasopressor-dependent shock states. Infection-related variables included preoperative Sequential Organ Failure Assessment (SOFA) score, preoperative norepinephrine infusion dose, and laboratory parameters reflecting inflammatory status and organ dysfunction. To further assess the severity of intra-abdominal infection and potential heterogeneity in baseline septic burden, the Mannheim Peritonitis Index (MPI) and primary intra-abdominal pathology were retrospectively calculated using perioperative clinical and intraoperative findings extracted from surgical and medical records. The MPI includes age, sex, organ failure, malignancy, duration of peritonitis, origin of sepsis, extent of peritonitis, and exudate characteristics, and has been validated as a prognostic indicator in patients with peritonitis and abdominal sepsis. In addition, diffuse peritonitis, fecal exudate, duration of peritonitis greater than 24 h, and primary intra-abdominal pathology were collected and analyzed. Primary intra-abdominal pathology was categorized as colonic ischemia/perforation, small bowel ischemia/perforation, gastroduodenal perforation, anastomotic leakage, complicated bowel obstruction, intra-abdominal abscess, and others. Surgical and anesthetic variables included surgery type, emergency status, duration of anesthesia and surgery, type and total dose of anesthetic agents used for induction and maintenance, and total doses of neuromuscular blocking agents and remifentanil.

To assess intraoperative hemodynamic stability, systolic blood pressure (SBP), diastolic blood pressure (DBP), MAP, HR, and peripheral oxygen saturation were recorded at six predefined time points: T1, baseline (before induction); T2, immediately after tracheal intubation; T3, at the start of surgery; T4, 15 min after surgical incision; T5, 30 min after surgical incision; and T6, at the end of surgery.

Outcome variables were selected to comprehensively evaluate the potential clinical impact of anesthetic choice on postoperative hemodynamic support, organ dysfunction, recovery trajectory, and major postoperative complications. The primary outcome was the Vasopressor-Inotrope Score (VIS) measured at ICU admission after surgery. VIS is a widely used index that quantifies the overall intensity of cardiovascular support and has demonstrated prognostic value in critically ill and surgical populations. Following the methods proposed by Gaies et al. and later evaluated in adult cohorts, VIS was calculated as follows (24–27):


V⁢I⁢S=dopamine⁢dose⁢(μ⁢g/kg/min)+dobutamine⁢dose

(μ⁢g/kg/min)+100×epinephrine⁢dose⁢(μ⁢g/kg/min)+

10×milrinonedose(μg/kg/min)+10,000×

vasopressindose(units/kg/min)+100×

norepinephrine⁢dose⁢(μ⁢g/kg/min)

Secondary outcomes included change in SOFA (ΔSOFA) at 24 h after ICU admission and Acute Physiology and Chronic Health Evaluation II (APACHE II) score as established indicators of organ dysfunction and illness severity, as well as mortality, ICU and hospital length of stay, mechanical ventilation duration, and major postoperative complications, which are commonly reported outcome measures in sepsis and critical care research (2, 5).

Laboratory values on postoperative days 1 and 2 included hemoglobin (g/dL), white blood cell count (×10^3^/μL), platelet count (×10^3^/μL), prothrombin time–international normalized ratio, activated partial thromboplastin time, albumin, aspartate aminotransferase, alanine aminotransferase, blood urea nitrogen, creatinine, sodium, potassium, chloride, lactate, and C-reactive protein (CRP).

### 2.4 Statistical analysis

Continuous variables are presented as mean (standard deviation), and categorical variables as counts (percentages). Between-group comparisons were performed using the Mann–Whitney U test for continuous variables and the chi-square test or Fisher’s exact test for categorical variables, as appropriate.

To reduce baseline imbalance between groups and approximate the conditions of a randomized design, propensity score matching (PSM) was performed. Propensity scores were estimated using a multivariable logistic regression model that included the following covariates: age, sex, BMI, ASA physical status, HTN, DM, total Charlson comorbidity index (CCI), baseline SOFA score, lactate level, and preoperative norepinephrine dose. Matching was conducted using a 1:1 nearest-neighbor algorithm with a caliper width of 0.25. Covariate balance in the matched cohort was considered acceptable when the standardized mean difference was <0.2. MPI, primary intra-abdominal pathology, peritonitis duration, diffuse generalized peritonitis, and exudate characteristics were not included in the propensity score model but were compared before and after matching to assess residual heterogeneity in intra-abdominal infection severity. Cases with incomplete medical records were excluded from the final analysis. Given the low proportion of missing data (5/238, 2.1%), analyses were performed using a complete-case approach without multiple imputation.

Major outcomes—including vasopressor requirement, intraoperative hemodynamic stability, ICU LOS, and 30-day mortality—were analyzed using the matched cohort. To further account for residual confounding after propensity score matching, post-matching multivariable regression analyses were additionally performed for postoperative outcomes. For continuous outcomes, general linear models were applied, whereas multivariable logistic regression was used for in-hospital mortality. All adjusted models consistently included operation time, age, body mass index, ASA physical status, hypertension, diabetes mellitus, CCI, baseline SOFA score, lactate level, preoperative norepinephrine dose, and MPI score as covariates reflecting patient characteristics, baseline septic severity, and intra-abdominal infection severity. Type III tests of model effects were used to evaluate the independent association between anesthetic group and postoperative outcomes after adjustment.

Longitudinal intraoperative hemodynamic changes were analyzed using linear mixed-effects models, with anesthetic group, time, and the group-by-time interaction specified as fixed effects and individual patients as random effects. Key demographic variables and baseline severity indicators were included as covariates. All statistical analyses were two-sided, and a *P*-value < 0.05 was considered statistically significant. Statistical analyses were performed using SPSS version 21 (SPSS Inc., Chicago, IL, USA).

## 3 Results

### 3.1 Patient characteristics

**FIGURE 1:**
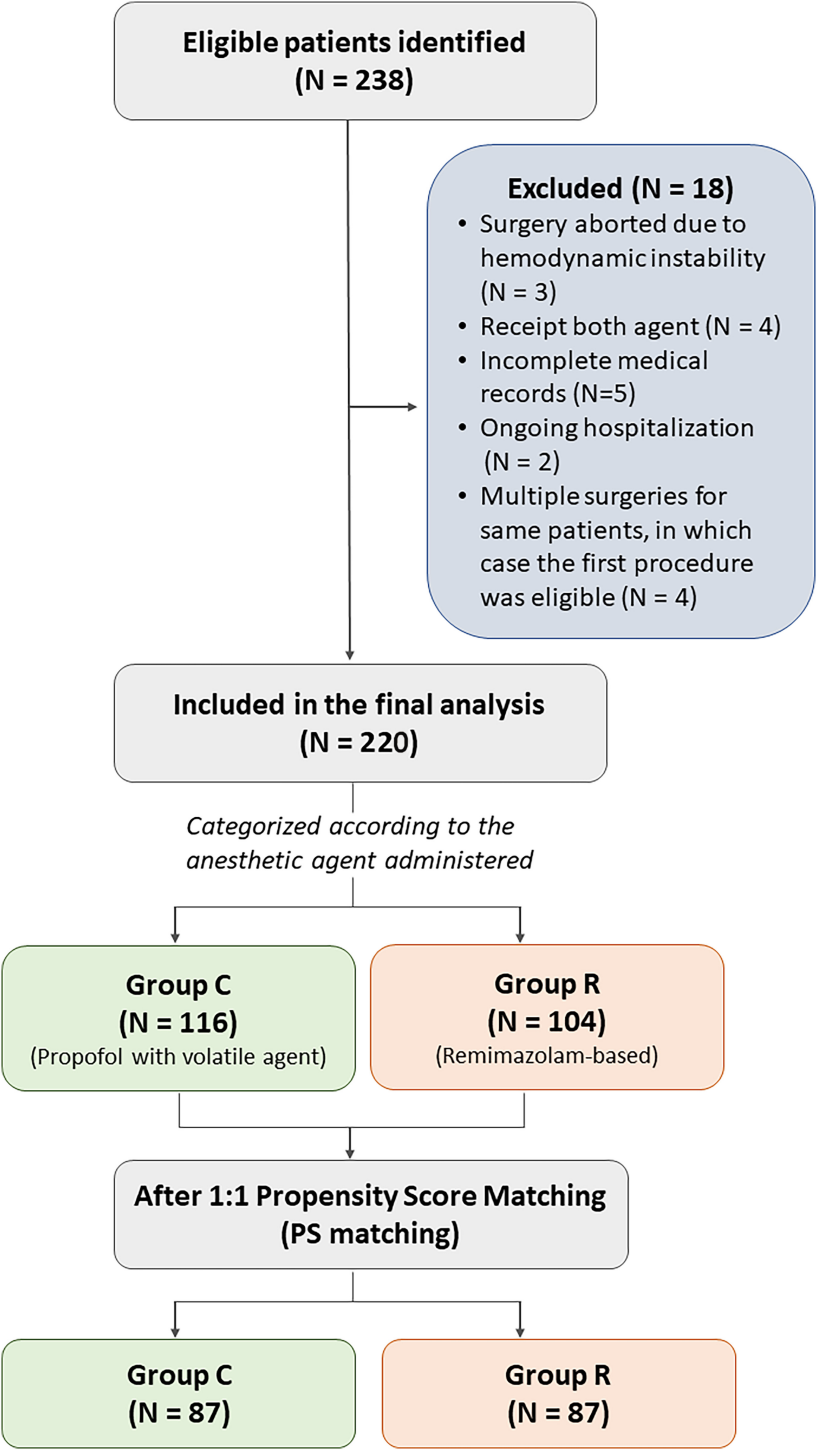
Flow diagram of patient selection and propensity score matching. Group C, propofol induction with volatile anesthetic maintenance; Group R, remimazolam-based total intravenous anesthesia. A total of 238 eligible patients were screened, of whom 220 were included in the final analysis (Group C, *n* = 116; Group R, *n* = 104). After 1:1 propensity score matching, 87 patients remained in each group.

Figure 1 presents the flow diagram of patient selection and cohort inclusion in the present study. Before matching, Group C and Group R included 116 and 104 patients, respectively. After PSM, 87 patients remained in each group. Baseline characteristics before and after propensity score matching are summarized in Table 1.

**TABLE 1:** Baseline and perioperative characteristics before and after propensity score matching.

Variable	Before matching			After matching		
	Group C (*n* = 116)	Group R (*n* = 104)	*P*-value	Group C (*n* = 87)	Group R (*n* = 87)	*P*-value
Age (years)	68.69 (14.33)	70.84 (12.56)	0.343	69.89 (13.82)	71.85 (12.14)	0.438
Sex, F, *n* (%)	44 (37.9)	43 (41.3)	0.605	33 (37.9)	33 (37.9)	1.000
BMI	21.78 (3.82)	22.27 (4.05)	0.351	21.93 (4.03)	21.86 (3.97)	0.906
Comorbidity						
HTN	67 (57.8)	59 (56.7)	0.878	52 (59.8)	52 (59.8)	1.000
DM	34 (29.3)	34 (32.7)	0.588	31 (35.6)	32 (36.8)	0.875
MI	3 (2.6)	7 (6.7)	0.197	3 (3.4)	6 (6.9)	0.304
CHF	11 (9.5)	13 (12.5)	0.474	6 (6.9)	12 (13.8)	0.135
CKD	14 (12.1)	11 (10.6)	0.728	11 (12.6)	8 (9.2)	0.466
Malignancy	76 (65.5)	51 (49.0)	0.014	57 (65.5)	44 (50.6)	0.056
ASA class	3.18 (0.67)	3.15 (0.52)	0.124	3.18 (0.67)	3.15 (0.52)	0.118
Total CCIs	3.86 (2.45)	3.78 (2.50)	0.817	3.87 (2.43)	3.78 (2.54)	0.769
Sepsis severity						
SOFA score	3.59 (3.56)	4.27 (3.76)	0.160	3.78 (3.63)	3.49 (3.07)	0.850
Lactate	3.70 (3.16)	2.88 (2.11)	0.136	3.05 (2.53)	2.86 (2.08)	0.946
Septic shock, *n* (%)	27 (23.3)	30 (28.8)	0.200	20 (23.0)	19 (21.8)	1.000
Preop NE dose	0.05 (0.14)	0.06 (0.12)	0.249	0.05 (0.14)	0.05 (0.12)	0.886
Surgical/infectious severity						
Diffuse peritonitis (%)	50 (43.1)	58 (55.8)	0.559	42 (48.3)	45 (51.7)	0.684
Fecal exudate (%)	25 (21.6)	29 (27.9)	0.559	21 (24.1)	23 (26.4)	0.344
Duration of peritonitis >24 h, *N* (%)	43 (37.1)	33 (31.7)		29 (33.3)	24 (27.6)	0.108
Total MPI score	23.54 (9.49)	24.90 (9.14)	0.200	23.49 (9.90)	23.93 (9.26)	0.733
Low risk (<21)	46 (39.7)	36 (34.6)	<0.001	34 (39.1)	35 (40.2)	0.941
Moderate risk (21–29)	38 (32.8)	34 (32.7)		28 (32.2)	28 (32.2)	
High risk (>29)	32 (27.6)	34 (32.7)		25 (28.7)	24 (27.6)	
Primary intra-abdominal pathology			0.032			0.181
Colonic ischemia/perforation	38 (32.8)	43 (41.3)		30 (34.5)	39 (44.8)	
Small bowel ischemia/perforation	26 (22.4)	15 (14.4)		16 (18.4)	12 (13.8)	
Gastroduodenal perforation	2 (1.7)	12 (11.5)		1 (1.1)	11 (12.6)	
Anastomotic leakage	6 (5.2)	6 (5.8)		5 (5.7)	4 (4.6)	
Complicated bowel obstruction	25 (21.6)	14 (13.5)		23 (26.4)	12 (13.8)	
Intra-abdominal abscess	8 (6.9)	5 (4.8)		6 (6.9)	4 (4.6)	
Others	11 (9.5)	9 (8.7)		6 (6.9)	5 (5.7)	
Preoperative laboratory findings						
Hemoglobin	10.75 (2.29)	11.29 (2.59)	0.032	10.75 (2.29)	11.59 (2.57)	0.304
WBC	11.35 (12.43)	11.06 (9.30)	0.763	11.67 (13.68)	10.96 (9.35)	0.563
PLT	238.84 (127.43)	221.18 (107.71)	0.557	243.62 (132.21)	236.05 (102.57)	0.864
Albumin	3.11 (0.71)	3.17 (0.66)	0.481	3.10 (0.71)	3.21 (0.66)	0.288
Creatinine	1.52 (1.51)	1.51 (1.33)	0.313	1.61 (1.70)	1.44 (1.27)	0.399
CRP	103.70 (100.12)	128.61 (92.01)	0.027	121.09 (97.08)	132.22 (96.09)	0.831

Values are presented as the mean (SD) or number of patients (%). Group C, propofol induction with volatile anesthetic maintenance; Group R, remimazolam-based total intravenous anesthesia. F, female; BMI, body mass index; HTN, hypertension; DM, diabetes mellitus; MI, myocardial infarction; CHF, congestive heart failure; CKD, chronic kidney disease; ASA, American Society of Anesthesiologists; CCI, Charlson comorbidity index; SOFA, Sequential Organ Failure Assessment; NE, norepinephrine; WBC, white blood cell count; PLT, platelet count; CRP, C-reactive protein.

In the matched cohort, demographic characteristics, comorbidities, baseline septic severity, and abdominal infectious burden were generally well balanced between the two groups. Indicators reflecting septic severity and abdominal infection severity, including SOFA score, serum lactate level, preoperative norepinephrine use and dose, total MPI score (23.49 ± 9.90 vs. 23.93 ± 9.26, *P* = 0.733), MPI risk category distribution (*P* = 0.941), diffuse generalized peritonitis (*P* = 0.684), fecal exudate (*P* = 0.344), and duration of peritonitis >24 h (*P* = 0.108), did not differ significantly between groups after matching. The distribution of primary intra-abdominal pathology differed significantly between groups before propensity score matching (*P* = 0.032); however, this imbalance was no longer significant after matching (*P* = 0.181).

Among preoperative laboratory findings, no clinically meaningful between-group differences were observed after matching. Detailed preoperative laboratory variables and types of surgery are provided in the Supplementary Tables 1, 2.

The proportion of emergency surgeries and the distribution of surgical procedures did not differ significantly between the two groups. However, operative time and anesthesia duration were longer in Group C than in Group R (operative time: 3.41 ± 1.95 h vs. 3.02 ± 1.52 h, *P* = 0.014; anesthesia time: 4.20 ± 2.10 h vs. 3.76 ± 1.41 h, *P* = 0.021) (Table 2). Except for the primary anesthetic agents (remimazolam and propofol), doses of adjunctive anesthetic agents, including Rocuronium and remifentanil, were generally comparable between groups and between the sepsis and septic shock subgroups.

**TABLE 2:** Intraoperative and postoperative data between groups.

	Group		
Variable	Group C (*n* = 87)	Group R (*n* = 87)	*P*-value
Intraoperative outcome			
Emergency, *n* (%)	74 (85.1)	80 (92.0)	0.154
Type of surgery, *n* (%)			0.354
Colorectal resection	37 (42.52)	28 (32.18)	
Small bowel and ileocecal surgery	24 (27.59)	30 (34.48)	
Other abdominal surgery	26 (29.88)	29 (33.33)	
OP time (h)	3.41 (1.95)	3.02 (1.52)	0.014
ANE time (h)	4.20 (2.10)	3.76 (1.414)	0.021
Remimazolam dose (mg/h)	0.00 (0.00)	54.25 (29.3)	<0.001
Sepsis	0.00 (0.00)	58.14 (39.63)	<0.001
Septic shock	0.00 (0.00)	50.73 (55.15)	<0.001
Propofol induction dose (mg)	75.57 (28.98)	0.00 (0.00)	<0.001
Sepsis	78.37 (29.48)	0.00 (0.00)	<0.001
Septic shock	67.78 (31.30)	0.00 (0.00)	<0.001
Rocuronium dose (mg/h)	36.52 (17.46)	34.80 (19.30)	0.076
Sepsis	38.49 (13.44)	37.46 (16.52)	0.150
Septic shock	35.72 (17.23)	33.62 (17.79)	0.061
Remifentanil dose (mg/h)	0.51 (3.12)	0.47 (0.71)	0.068
Sepsis	0.62 (3.12)	0.64 (3.12)	0.142
Septic shock	0.29 (0.26)	0.34 (0.24)	0.081
Intraop input			
Crystalloid (ml/h)	616.69 (384.30)	618.39 (398.98)	0.576
Colloid (ml/h)	8.74 (24.10)	7.99 (36.75)	0.051
Albumin (ml/h)	49.13 (87.31)	57.09 (102.46)	0.649
RBC (units)	1.03 (2.65)	0.84 (1.21)	0.056
FFP (units)	0.63 (1.72)	0.26 (0.83)	0.068
Apheresis (units)	0.34 (2.24)	0.03 (0.24)	0.150
Intraop output			
Urine (ml/h)	91.29 (82.81)	99.25 (98.57)	0.042
Blood (ml/h)	133.25 (446.35)	127.24 (469.09)	0.058
Postoperative outcome			
Vasopressor requirements (VIS)	10 (0, 29.5)	6 (0, 20)	0.008
Sepsis	5 (0, 20)	3 (0, 17)	0.014
Septic shock	27 (18.5, 54.95)	15 (8, 36.5)	0.007
ΔSOFA (24 h ICU-adm)	5.34 (3.77)	3.97 (4.32)	0.021
Sepsis	6.15 (4.14)	3.88 (4.45)	0.001
Septic shock	4.41 (3.31)	3.17 (3.49)	0.174
ICU LOS	6.74 (9.36)	4.18 (5.91)	0.008
Hospital LOS	26.34 (21.46)	18.55 (11.52)	0.012
Mechanical ventilation days	2.15 (4.71)	1.74 (3.75)	0.178
30-day mortality	21 (24.1)	14 (16.1)	0.186
In-hospital mortality	33 (37.9)	17 (19.5)	0.007
APACHE II scores	27.54 (10.66)	25.11 (10.08)	0.162
AKI	36 (41.4)	24 (27.6)	0.056
ARDS	5 (5.7)	4 (4.6)	0.732
Delirium	39 (44.8)	31 (35.6)	0.216
Clavien–Dindo classification ≥3	42 (48.3)	30 (34.5)	0.065
Hospital-acquired infections	48 (55.2)	35 (40.2)	0.048
ICU readmission rates	10 (11.6)	8 (9.2)	0.600

Values are presented as the mean (SD), median (IQR), or number of patients (%). Group C, propofol induction with volatile anesthetic maintenance; Group R, remimazolam-based total intravenous anesthesia. OP, operative time; ANE, anesthesia time; RBC, red blood cells; FFP, fresh frozen plasma; SOFA, Sequential Organ Failure Assessment; ICU, intensive care unit; LOS, length of stay; APACHE, Acute Physiology and Chronic Health Evaluation II; AKI, acute kidney injury; ARDS, acute respiratory distress syndrome.

As expected, anesthetic exposure differed between groups. Group R received remimazolam at a mean infusion rate of 54.25 ± 29.3 mg/h, whereas Group C received propofol as a mean induction dose of 75.57 ± 28.98. In subgroup analyses according to baseline septic severity, remimazolam and propofol exposure remained consistent within the sepsis and septic shock subgroups, whereas doses of adjunctive anesthetic agents, including Rocuronium and remifentanil normalized to anesthesia duration, did not differ significantly between groups. Intraoperative fluid administration, blood product transfusion, and estimated blood loss were comparable between the groups. However, hourly urine output was slightly lower in Group C (Table 2).

### 3.2 Postoperative outcomes

The primary outcome, vasopressor requirement at postoperative ICU admission, was significantly lower in Group R than in Group C (Table 2; 6 [0–20] vs. 10 [0–29.5], *P* = 0.008) in both the sepsis subgroup (3 [0–17] vs. 5 [0–20], *P* = 0.014) and the septic shock subgroup (15 [8–36.5] vs. 27 [18.5–54.95], *P* = 0.007). Mean ± SD values of VIS, as well as patient-level disaggregation of vasopressor and inotrope exposure, are additionally presented in Supplementary Table 3. The ΔSOFA score at 24 h after ICU admission was significantly lower in Group R than in Group C (3.97 ± 4.32 vs. 5.34 ± 3.77, *P* = 0.021) (Table 2). In subgroup analysis, this difference remained significant in patients with sepsis (3.88 ± 4.45 vs. 6.15 ± 4.14, *P* = 0.001), but not in those with septic shock (*P* = 0.174). Indicators of clinical recovery also favored Group R. Patients in Group R had shorter ICU LOS (4.18 ± 5.91 days vs. 6.74 ± 9.36 days, *P* = 0.008) and overall hospital LOS (18.55 ± 11.52 days vs. 26.34 ± 21.46 days, *P* = 0.012).

There were no significant between-group differences in duration of mechanical ventilation or 30-day mortality. However, in-hospital mortality and incidence of hospital-acquired infections were lower in Group R (19.5% vs. 37.9%, *P* = 0.007; 40.2% vs. 55.2%, *P* = 0.048, respectively). No significant between-group differences were observed in the incidence of AKI, ARDS, delirium, Clavien–Dindo grade ≥III complications, or ICU readmission rates (Table 2). The ΔSOFA at 24 h was significantly smaller in Group R than in Group C (3.97 ± 4.32 vs. 5.34 ± 3.77, *P* = 0.021) (Table 2). Postoperative laboratory findings are shown in Supplementary Table 4, with no significant differences between the groups except POD 1 sodium, POD 2 hemoglobin, potassium.

### 3.3 Intraoperative hemodynamic parameters

**FIGURE 2:**
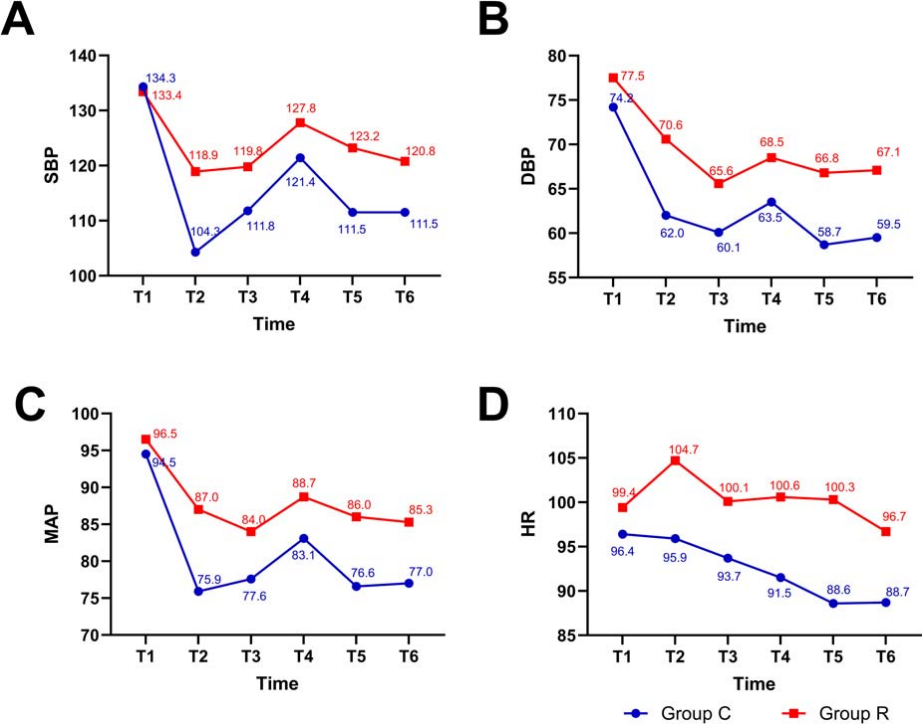
Intraoperative hemodynamic changes according to anesthetic group. Group C, propofol induction with volatile anesthetic maintenance; Group R, remimazolam-based total intravenous anesthesia. Mixed-effects model analysis demonstrated significant group-by-time interactions for systolic blood pressure (SBP, **A**, *P* = 0.024) and mean arterial pressure (MAP, **C**, *P* = 0.049), indicating different temporal patterns between groups, with higher SBP and MAP maintained in the remimazolam group. Diastolic blood pressure (DBP, **B**) showed significant main effects of time (*P* < 0.001) and group (*P* < 0.001) but no significant group-by-time interaction (*P* = 0.181). Heart rate (HR, **D**) demonstrated significant effects of time (*P* < 0.001) and group (*P* = 0.002), with a significant group-by-time interaction (*P* < 0.001).

Intraoperative SBP and MAP demonstrated a significant group-by-time interaction (SBP: *P* = 0.024, MAP: *P* = 0.049) (Figure 2). Throughout the intraoperative period, SBP and MAP tended to remain at higher levels in Group R, consistent with greater hemodynamic stability. DBP showed significant main effects of time (*P* < 0.001) and anesthetic group (*P* < 0.001); however, the group-by-time interaction was not statistically significant (*P* = 0.181). HR demonstrated significant effects of time (*P* < 0.001) and anesthetic group (*P* = 0.002), as well as significant group-by-time interaction (*P* < 0.001).

### 3.4 Adjusted analyses after propensity score matching

To further account for residual confounding after propensity score matching, post-matching multivariable regression analyses were additionally performed for postoperative outcomes (Table 3). After additional adjustment including operative time, age, BMI, ASA physical status, baseline SOFA score, lactate level, and preoperative norepinephrine use, and MPI score, anesthetic group remained independently associated with postoperative vasopressor requirement (*F* = 3.71, *P* = 0.046), ICU length of stay (*F* = 4.93, *P* = 0.028), hospital length of stay (*F* = 3.94, *P* = 0.049), and in-hospital mortality (χ^2^ = 6.90, *P* = 0.009). In contrast, the association between anesthetic group and ΔSOFA at 24 h was no longer statistically significant after adjustment (*F* = 2.53, *P* = 0.114). These associations remained significant despite additional adjustment for operative factors, baseline septic severity, comorbidity burden, preoperative vasopressor requirement, and intra-abdominal infection severity represented by MPI score, supporting the robustness of the observed group effect.

**TABLE 3:** Post-matching multivariable regression analyses of postoperative outcomes.

Outcome	Group effect	*P*
Vasopressor requirement	*F* = 3.71	0.046
ΔSOFA at 24 h	*F* = 2.53	0.114
ICU LOS	*F* = 4.93	0.028
Hospital LOS	*F* = 3.94	0.049
In-hospital mortality	χ2 = 6.90	0.009

All adjusted models included operation time, age, body mass index, ASA physical status, hypertension, diabetes mellitus, Charlson comorbidity index, baseline SOFA score, lactate level, preoperative norepinephrine dose, and Mannheim Peritonitis Index score as predefined covariates. OP, operative time; BMI, body mass index; ASA, American Society of Anesthesiologists; SOFA, Sequential Organ Failure Assessment; NE, norepinephrine; ICU, intensive care unit; LOS, length of stay.

## 4 Discussion

In this study, remimazolam-based anesthesia in patients with sepsis or septic shock was associated with more stable intraoperative hemodynamic profiles, lower postoperative vasopressor requirements, and shorter ICU and hospital LOS compared with propofol-based balanced anesthesia. These associations largely remained after adjustment for operative factors, baseline septic severity, comorbidity burden, preoperative vasopressor requirement, and intra-abdominal infection severity including MPI score. Although in-hospital mortality was lower in the remimazolam group, 30-day mortality did not differ significantly; therefore, this finding should be interpreted cautiously, as the shorter hospital LOS may have influenced the opportunity to capture in-hospital deaths.

The reduction in postoperative vasopressor requirement may be clinically meaningful because vasopressor exposure is associated with impaired regional perfusion, increased myocardial oxygen demand, and microcirculatory dysfunction. Therefore, the hemodynamic advantages of remimazolam may have implications beyond the prevention of transient hypotension and potentially influence postoperative recovery. To our knowledge, this study is among the first to evaluate not only intraoperative hemodynamic outcomes but also postoperative vasopressor requirements and recovery-related outcomes in patients with sepsis or septic shock.

In patients with sepsis or septic shock, arterial tone is typically reduced, and autonomic dysregulation, impaired vascular responsiveness, and microcirculatory dysfunction are common (28, 29). Additional vasodilation or myocardial depression during anesthetic induction and maintenance may further destabilize an already fragile hemodynamic state. Because patients with abdominal sepsis frequently require urgent surgery for source control, minimizing intraoperative hemodynamic fluctuations and avoiding excessive fluid administration or high-dose vasopressor exposure are particularly important during the early perioperative phase.

In clinical practice, etomidate and ketamine are often considered as induction agents in patients with sepsis because of their relative cardiovascular stability. Etomidate is favored for its minimal immediate hemodynamic effects; however, concerns remain regarding its suppression of adrenal steroidogenesis, even after a single induction dose. This effect may impair stress response and has been associated with worse outcomes in septic shock in several meta-analyses (30–32). Ketamine, on the other hand, provides sympathetic stimulation and may transiently support arterial pressure. Nevertheless, in patients with prolonged or severe septic shock characterized by catecholamine depletion, its cardiovascular effects may be unpredictable, and potential increases in myocardial oxygen consumption or arrhythmogenicity may limit its clinical utility (33, 34). In this context, remimazolam may represent an alternative strategy, offering hemodynamic stability without adrenal suppression and allowing more predictable titration during both induction and maintenance of anesthesia.

The observed hemodynamic stability and reduced postoperative vasopressor requirements in Group R may be explained by the pharmacodynamic properties of remimazolam. These findings are consistent with recent reports suggesting that remimazolam may offer favorable cardiovascular tolerance in critically ill patients while preserving predictable anesthetic depth and recovery characteristics (9, 35). Compared with balanced anesthesia, remimazolam has been associated with smaller reductions in MAP during induction and maintenance, as well as reduced vasopressor requirements (21, 29). Similar findings have been observed in high-risk populations, including patients with ASA physical status III or higher and those undergoing cardiac surgery (19, 22). These effects are thought to be attributable to the relatively limited vasodilatory and myocardial depressant properties of remimazolam (22, 29, 36). Importantly, most previous studies have focused primarily on short-term hemodynamic outcomes, such as hypotension, vasopressor use, and blood pressure variability during anesthesia. In contrast, the present study evaluated both intraoperative hemodynamic trajectories and clinically relevant postoperative outcomes in patients with sepsis or septic shock, a population in whom evidence regarding remimazolam remains limited.

In septic condition, excessive vasopressor load has been associated with increased myocardial oxygen demand and ischemia, maldistribution of regional blood flow—particularly in peripheral and splanchnic beds—worsening microcirculatory heterogeneity, peripheral ischemia or necrosis, and increased mortality risk (16, 18, 37). The lower vasopressor requirements and shorter LOS in Group R suggest that anesthetic choice may influence not only intraoperative hemodynamic burden but also the overall trajectory of postoperative recovery. However, early changes in organ dysfunction (ΔSOFA) were largely explained by baseline severity, indicating that remimazolam may confer indirect clinical benefits primarily through improved hemodynamic stability rather than direct modification of sepsis pathophysiology. Moreover, a 24-h observation period may be insufficient to capture the full trajectory of organ function recovery. In contrast, cumulative and integrative outcome measures—such as vasopressor requirement, ICU and hospital LOS, and in-hospital mortality—continued to demonstrate significant between-group differences. This pattern suggests that the potential effects of remimazolam may be more accurately reflected in the broader postoperative course rather than in early organ dysfunction scores. To our knowledge, few previous studies have examined whether the hemodynamic advantages of remimazolam translate into downstream recovery-related outcomes in critically ill surgical patients. Therefore, the observed associations with ICU length of stay, hospital length of stay, and in-hospital mortality may provide clinically relevant insights extending beyond traditional hemodynamic endpoints.

This study has several limitations. First, as an observational study, selection bias and residual confounding could not be entirely excluded. In particular, the exact interval between sepsis onset and surgical intervention could not be standardized because of the retrospective design, which may have contributed to heterogeneity in inflammatory status and disease progression among patients. In addition, because anesthetic agent selection and vasopressor initiation were determined at the discretion of the attending clinicians, practice variation and selection bias may have influenced perioperative management and outcomes. Although SOFA score, lactate level, MPI score, and primary intra-abdominal pathology were generally comparable between groups after propensity score matching, unmeasured confounders may still have influenced the results. In addition, although in-hospital mortality was lower in the remimazolam group, 30-day mortality did not significantly differ between groups. Because hospital LOS was also shorter in the remimazolam group, time-at-risk bias related to earlier discharge may have partially influenced the opportunity to capture in-hospital deaths. Therefore, the observed difference in in-hospital mortality should be interpreted cautiously. Second, the vasopressor requirement at ICU admission was used as a pragmatic surrogate; however, this measure may not completely represent total intraoperative vasopressor exposure. Accordingly, interpretations regarding vasopressor burden should be approached cautiously. Third, although the proportion of missing data was small, missing or incompletely recorded variables were unavoidable in this retrospective study. Therefore, the possibility of residual bias related to missing data cannot be entirely excluded. Finally, the single-center design and the small sample size may have limited statistical power and generalizability, particularly rare complications or subgroup analyses. Future multicenter or prospective studies are warranted to validate and extend these findings.

Despite these limitations, remimazolam-based anesthesia may represent a reasonable option for patients with preexisting hemodynamic instability, those requiring preoperative vasopressor support, or those at high risk for intraoperative hypotension. By attenuating intraoperative hemodynamic variability and reducing postoperative vasopressor requirements, remimazolam may contribute to more favorable postoperative resource utilization and recovery trajectories. Prospective randomized trials are needed to clarify the causal impact of remimazolam-based anesthesia on clinical outcomes in this high-risk population.

## 5 Conclusion

In patients with sepsis or septic shock undergoing abdominal source-control surgery, remimazolam-based anesthesia was associated with greater intraoperative hemodynamic stability, reduced postoperative vasopressor requirements, shorter ICU and hospital length of stay, and lower in-hospital mortality compared with propofol-based balanced anesthesia. These findings suggest that anesthetic strategies aimed at minimizing cardiovascular depression may have clinically relevant implications beyond the prevention of transient intraoperative hypotension and may contribute to improved postoperative recovery in this high-risk population. Given the observational nature of the present study, prospective randomized trials are warranted to determine whether these associations reflect a causal benefit of remimazolam-based anesthesia on postoperative outcomes.

## Data Availability

The original contributions presented in this study are included in this article/Supplementary material, further inquiries can be directed to the corresponding author.

## Supplementary material

Supplementary Table 1 Detailed types of surgery between groups.

Supplementary Table 2 Detailed preoperative laboratory findings between groups.

Supplementary Table 3 Patient-level disaggregation of vasopressor and inotrope exposure at ICU admission after propensity score matching.

Supplementary Table 4 Comparison of postoperative laboratory findings between groups.
