# Supplementary material for: Remimazolam-based anesthesia in sepsis patients: a retrospective cohort study of hemodynamic stability and postoperative outcomes
Source: Front Med (Lausanne). 2026 Jul 1;13:1838670. doi: 10.3389/fmed.2026.1838670 (PMC13369609; doi:10.3389/fmed.2026.1838670)
Supplement: Supplementary Table 1 — Detailed types of surgery between groups. [file Supplementary_file_1.docx]

**Supplemental table 1**. Detailed types of surgery between groups.

|  | Group | |  |
| --- | --- | --- | --- |
|  | Group C (n=87) | Group R (n=87) | P-value |
| **Type of surgery, n (%)** |  |  | 0.354 |
| **Colorectal Resection** | 37 (42.52) | 28 (32.18) |  |
| Abdominoperineal resection | 1 ( 1.1) | 0 ( 0.0) |  |
| Anterior resection of sigmoid colon | 2 ( 1.1) | 4 ( 2.3) |  |
| Right hemicolectomy | 7 ( 5.7) | 5 ( 5.7) |  |
| Segmental resection of colon | 4 ( 2.3) | 1 ( 0.0) |  |
| Subtotal colectomy | 2 ( 2.3) | 3 ( 3.4) |  |
| Total colectomy | 4 ( 4.6) | 3 ( 3.4) |  |
| Total proctocolectomy with end ileostomy | 0 ( 0.0) | 2 ( 1.1) |  |
| Hartmann operation | 16 (18.4) | 10 (11.5) |  |
| Revision of anastomosis of large intestine | 1 ( 1.1) | 0 ( 0.0) |  |
| **Small bowel & ileocecal surgery** | 24 (27.59) | 30 (34.48) |  |
| Small bowel resection | 20 (21.8) | 13 (14.9) |  |
| Ileocecectomy | 2 ( 2.3) | 13 ( 2.3) |  |
| Partial gastrectomy | 0 ( 0.0) | 1 ( 1.1) |  |
| Loop ileostomy | 1 ( 1.1) | 3 ( 3.4) |  |
| Revision of ileostomy | 1 ( 1.1) | 0 ( 0.0) |  |
| **Other abdominal surgery** | 26 (29.88) | 29 (33.33) |  |
| Exploratory laparotomy | 17 (19.5) | 18 (20.7) |  |
| Adhesiolysis of abdomen | 7 ( 8.0) | 3 ( 2.3) |  |
| Primary closure of duodenal ulcer | 0 ( 0.0) | 4 ( 4.6) |  |
| Primary closure of gastric ulcer | 0 ( 0.0) | 4 ( 4.6) |  |
| Primary repair of colon | 2 ( 2.3) | 0 ( 0.0) |  |

Values are presented as number of patients (%).

**Supplemental table 2.** Detailed preoperative laboratory findings between groups.

|  | **Before matching** | |  | **After matching** | |  |
| --- | --- | --- | --- | --- | --- | --- |
|  | **Group C (n=116)** | **Group B (n=104)** | **P-value** | **Group C (n=78)** | **Group B (n=78)** | **P-value** |
| Hemoglobin | 10.75 (2.29) | 11.29 (2.59) | 0.032 | 10.75 (2.29) | 11.59 (2.57) | 0.304 |
| WBC | 11.35 (12.43) | 11.06 (9.30) | 0.763 | 11.67 (13.68) | 10.96 (9.35) | 0.563 |
| PLT | 238.84 (127.43) | 221.18 (107.71) | 0.557 | 243.62 (132.21) | 236.05 (102.57) | 0.864 |
| PT INR | 1.24 (0.33) | 1.29 (0.33) | 0.083 | 1.19 (0.28) | 1.26 (0.30) | 0.102 |
| aPTT | 41.11 (17.04) | 39.45 (9.27) | 0.798 | 39.97 (11.76) | 39.05 (9.51) | 0.607 |
| Protein | 5.93 (1.16) | 5.95 (1.00) | 0.899 | 5.89 (1.21) | 6.01 (0.97) | 0.490 |
| Albumin | 3.11 (0.71) | 3.17 (0.66) | 0.481 | 3.10 (0.71) | 3.21 (0.66) | 0.288 |
| AST | 93.99 (590.36) | 53.62 (126.51) | 0.452 | 107.24 (680.18) | 53.11 (135.29) | 0.433 |
| ALT | 38.52 (134.26) | 31.18 (68.31) | 0.859 | 32.17 (98.57) | 31.29 (73.30) | 0.791 |
| Glucose | 140.99 (57.95) | 153.74 (83.74) | 0.241 | 145.25 (61.59) | 156.47 (88.50) | 0.487 |
| BUN | 31.21 (23.73) | 30.51 (19.85) | 0.589 | 32.66 (26.39) | 28.82 (18.47) | 0.963 |
| Creatinine | 1.52 (1.51) | 1.51 (1.33) | 0.313 | 1.61 (1.70) | 1.44 (1.27) | 0.399 |
| Na | 137.00 (4.36) | 135.57 (5.31) | 0.031 | 136.15 (4.34) | 135.30 (5.04) | 0.090 |
| K | 5.81 (19.04) | 4.01 (0.56) | 0.694 | 3.98 (0.71) | 4.00 (0.53) | 0.596 |
| Cl | 102.12 (10.64) | 101.75 (5.68) | 0.116 | 101.83 (11.96) | 101.74 (5.42) | 0.138 |
| Lactate | 3.70 (3.16) | 2.88 (2.11) | 0.136 | 3.05 (2.53) | 2.86 (2.08) | 0.946 |
| CRP | 103.70 (100.12) | 128.61 (92.01) | 0.027 | 121.09 (97.08) | 132.22 (96.09) | 0.831 |

Data are expressed as mean (SD) or number (frequency). WBC, white blood cell count; PLT, platelet count; PT-INR, prothrombin time–international normalized ratio; aPTT, activated partial thromboplastin time; AST, aspartate aminotransferase; ALT, alanine aminotransferase; BUN, blood urea nitrogen; Na, sodium; K, potassium; Cl, chloride; CRP, C-reactive protein.

**Supplemental table 3**. Patient-level disaggregation of vasopressor and inotrope exposure at ICU admission after propensity score matching

| **Variable** | **Group C**  **(n = 87)** | **Group R**  **(n = 87)** | **P value** |
| --- | --- | --- | --- |
| **Overall VIS** |  |  |  |
| Overall VIS, mean ± SD | 18.50 ± 22.38 | 10.63 ± 15.58 | 0.006 |
| **Norepinephrine exposure** |  |  |  |
| Patients receiving norepinephrine, n (%) | 58 (66.7) | 51 (58.6) | 0.273 |
| Norepinephrine dose (μg/kg/min) | 0.2 (0.1–0.3) | 0.16 (0.05–0.3) |  |
| **Epinephrine exposure** |  |  |  |
| Patients receiving epinephrine, n (%) | 9 (10.3) | 11 (12.6) | 0.635 |
| Epinephrine dose (μg/kg/min) | 0.15 (0.09–0.2) | 0.05 (0.02–0.1) |  |
| **Vasopressin exposure** |  |  |  |
| Patients receiving vasopressin, n (%) | 14 (16.1) | 13 (14.9) | 0.834 |
| Vasopressin dose (units/kg/min) | 0.00056  (0.0003–0.0007) | 0.00039  (0.0002–0.0005) |  |
| **Dobutamine exposure** |  |  |  |
| Patients receiving dobutamine, n (%) | 1 (1.1) | 0 (0) | 1 |
| Dobutamine dose (μg/kg/min) | 4 (4–4) | N/A |  |
| **Dopamine exposure** |  |  |  |
| Patients receiving dopamine, n (%) | 0 (0) | 0 (0) | 1 |

Values are presented as median (IQR), mean ± SD, or number of patients (%), as appropriate.
VIS, vasopressor–inotropic score.

**Supplemental table 4.** Comparison of postoperative laboratory findings between groups.

|  | Group | |  |  |
| --- | --- | --- | --- | --- |
|  | Group C (n=87) | Group R (n=87) | P-value |  |
| **POD 1 lab findings** |  |  |  | |
| Hemoglobin | 9.98 (6.96) | 10.02 (1.93) | 0.053 | |
| WBC | 11.27 (6.49) | 10.12 (7.08) | 0.096 | |
| PLT | 172.81 (108.08) | 190.18 (102.11) | 0.129 | |
| PT INR | 1.52 (0.30) | 1.56 (0.64) | 0.797 | |
| aPTT | 48.54 (18.35) | 46.62 (11.53) | 0.754 | |
| Albumin | 2.73 (0.38) | 2.76 (0.38) | 0.374 | |
| AST | 78.56 (297.84) | 138.79 (692.05) | 0.612 | |
| ALT | 33.10 (114.61) | 53.80 (208.85) | 0.350 | |
| BUN | 28.05 (21.11) | 25.90 (16.58) | 0.869 | |
| Creatinine | 1.41 (1.34) | 1.24 (0.98) | 0.928 | |
| Na | 139.68 (3.71) | 137.71 (4.33) | 0.002 | |
| K | 3.73 (0.64) | 3.83 (0.56) | 0.378 | |
| Cl | 105.13 (12.00) | 105.92 (4.35) | 0.840 | |
| Lactate | 2.54 (1.90) | 2.29 (2.29) | 0.376 | |
| CRP | 141.66 (69.29) | 166.88 (65.01) | 0.185 | |
| **POD 2 lab findings** |  |  |  | |
| Hemoglobin | 8.68 (1.37) | 9.38 (1.84) | 0.035 | |
| WBC | 11.59 (6.93) | 9.87 (5.56) | 0.084 | |
| PLT | 152.19 (110.46) | 175.13 (110.79) | 0.061 | |
| PT-INR | 1.45 (0.33) | 1.52 (0.46) | 0.479 | |
| aPTT | 52.28 (17.56) | 49.61 (11.81) | 0.147 | |
| Albumin | 2.70 (0.32) | 2.73 (0.40) | 0.839 | |
| AST | 103.16 (440.21) | 242.94 (1184.69) | 0.910 | |
| ALT | 55.02 (216.97) | 96.74 (367.26) | 0.846 | |
| BUN | 23.69 (18.18) | 23.70 (16.75) | 0.739 | |
| Creatinine | 1.17 (1.08) | 1.16 (1.02) | 0.751 | |
| Na | 139.74 (4.02) | 138.70 (3.26) | 0.145 | |
| K | 3.48 (0.58) | 4.84 (10.40) | 0.008 | |
| Cl | 106.05 (4.45) | 105.27 (11.91) | 0.568 | |
| Lactate | 2.12 (2.88) | 2.04 (2.93) | 0.931 | |
| CRP | 165.59 (60.28) | 172.09 (78.27) | 0.702 | |

Data are expressed as mean (SD) or number (frequency). WBC, white blood cell count; PLT, platelet count; PT-INR, prothrombin time–international normalized ratio; aPTT, activated partial thromboplastin time; AST, aspartate aminotransferase; ALT, alanine aminotransferase; BUN, blood urea nitrogen; Na, sodium; K, potassium; Cl, chloride; CRP, C-reactive protein.
